# Amplified therapeutic targets in high-grade serous ovarian carcinoma – a review of the literature with quantitative appraisal

**DOI:** 10.1038/s41417-023-00589-z

**Published:** 2023-02-20

**Authors:** Thomas Talbot, Haonan Lu, Eric O. Aboagye

**Affiliations:** Department of Surgery & Cancer, Imperial College London, Hammersmith Hospital, Du Cane Road, W120NN London, UK

**Keywords:** Oncogenes, Ovarian cancer

## Abstract

High-grade serous ovarian carcinoma is a unique cancer characterised by universal *TP53* mutations and widespread copy number alterations. These copy number alterations include deletion of tumour suppressors and amplification of driver oncogenes. Given their key oncogenic roles, amplified driver genes are often proposed as therapeutic targets. For example, development of anti-HER2 agents has been clinically successful in treatment of *ERBB2*-amplified tumours. A wide scope of preclinical work has since investigated numerous amplified genes as potential therapeutic targets in high-grade serous ovarian carcinoma. However, variable experimental procedures (e.g., choice of cell lines), ambiguous phenotypes or lack of validation hinders further clinical translation of many targets. In this review, we collate the genes proposed to be amplified therapeutic targets in high-grade serous ovarian carcinoma, and quantitatively appraise the evidence in support of each candidate gene. Forty-four genes are found to have evidence as amplified therapeutic targets; the five highest scoring genes are *CCNE1*, *PAX8*, *URI1*, *PRKCI* and *FAL1*. This review generates an up-to-date list of amplified therapeutic target candidates for further development and proposes comprehensive criteria to assist amplified therapeutic target discovery in the future.

## Introduction

Ovarian cancer (OC) is a significant cause of global female mortality. Epithelial ovarian cancer (EOC) represents over 90% of cases, with high-grade serous ovarian carcinoma (HGSOC) being the most common (70% of EOC) and deadly subtype due to its predilection for recurrence after initial treatment [1]. HGSOC is recognised as a copy number driven cancer (CNDC): it is characterised by chromosomal instability (CIN) leading to widespread regions of genomic loss and gain [2, 3]. Pathogenic *TP53* mutations are near universal in HGSOC [4], with loss of p53 function understood to be a key early event in HGSOC tumorigenesis leading to CIN, often provoked further by homologous recombination deficiency (HRD). Gene mutations in HGSOC besides *TP53* and *BRCA1/2* (associated with hereditary OC and HRD) are rare (mutation prevalences of 2–6% in HGSOC specimens) in comparison to other cancers [2, 3]. Other cancer types known to be associated with frequent copy number alterations (CNA) include a subset of breast and oesophageal cancers [5]. Some cancers, e.g., those with microsatellite instability, have lower frequencies of CNA and instead have higher mutational burden [6]. CNAs and gene mutations are not mutually exclusive however; [3] there is instead a spectrum of changes that occur across cancers. Whilst the study of activating oncogene mutations has permitted development of efficacious drugs (e.g., vemurafenib for *BRAF* V600E melanoma) [7], use and development of targeted drugs for CNDCs has proven more challenging. Correspondingly, overall survival (OS) in HGSOC has not significantly improved over the last 20 years [8]. Poly ADP-ribose polymerase (PARP) inhibitors, such as olaparib, are newer approvals showing clinical success, and have promise in improving disease survival. In a phase 3 randomised clinical trial for maintenance treatment of platinum-sensitive relapsed OC with a BRCA1/2 mutation, olaparib achieved a progression-free survival (PFS) improvement of 13.6 months relative to placebo [9]. In a final analysis, unadjusted for patients in the placebo arm later receiving olaparib, olaparib yielded an OS benefit of 12.9 months compared to placebo, although statistical significance was not reached [10]. Rucaparib, another PARP inhibitor, improved PFS in a phase 3 randomised placebo-controlled clinical trial in the maintenance setting for recurrent OC after response to platinum-based chemotherapy: 9.2 months benefit in patients with BRCA-mutations and 5.4 months in the intention-to-treat population, including patients without BRCA-mutations or HRD [11]. Niraparib, another PARP inhibitor, also improved PFS in a phase 3 randomised placebo-controlled trial for relapsed OC, with a benefit of 15.5 months in germline-BRCA mutant OC and 5.4 months in the overall non-germline-BRCA mutant cohort [12]. Niraparib also demonstrated improved PFS as maintenance treatment after first-line platinum-based chemotherapy, with a benefit of 11.5 months in the HRD group and 5.6 months in the overall population [13]. PARP inhibitors exploit synthetic lethality in cancers with HRD rather than amplified genes themselves [14].

**Fig. 1:**
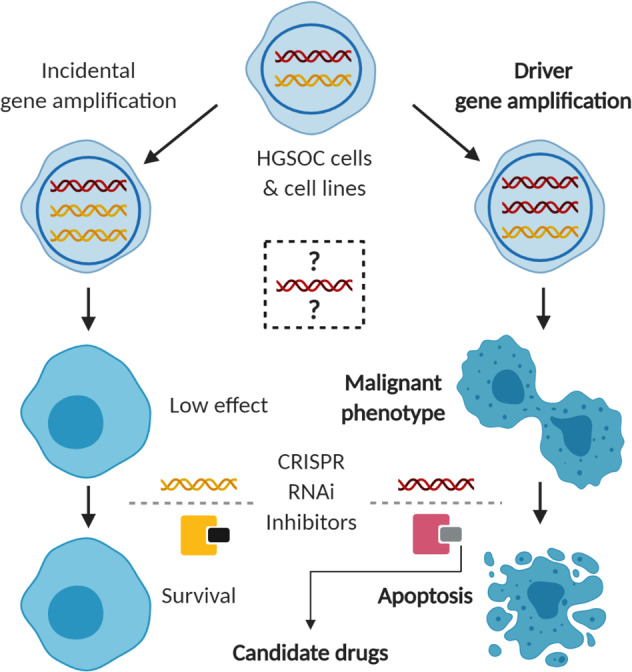
Schematic of knockdown experiments used to validate amplified therapeutic targets in high-grade serous ovarian carcinoma. Knockdown of bystander genes or inhibition of their protein products has little to no effect on cell survival, whereas knockdown of amplified driver genes suppresses the malignant phenotype with translational potential in the clinic.

Within CNDCs, clusters of amplified genes at restricted regions of the genome, referred to as amplicons, can vary greatly in both size and genome location between tumours of the same type. Amplicons may contain numerous genes (the commonly amplified 20q locus may contain 132 genes [15]) but only one or a few of these genes engender a malignant phenotype: these are the amplified driver genes [16]. Other amplified genes may not carry a malignant phenotype, or not be overexpressed [17]. Since CNDCs depend on overexpression of driver genes for malignant behaviour, RNA-mediated knockdown (KD) or CRISPR/Cas9-mediated knockout of amplified drivers causes loss of cancer phenotype. These are some of the core experiments used to validate their role and may infer therapeutic potential (Fig. 1) (ref. [17, 18]). Recently, copy number profiles from 132 patient samples have been modelled into seven copy number signatures, reflecting the different underlying genomic aberrations in HGSOC, including breakage-fusion-bridge cycles, tandem duplication and chromothripsis. Nearly all HGSOCs display multiple signatures concurrently [8], reflecting the significant intertumoral heterogeneity seen in the disease. Intratumoral heterogeneity is also prevalent: spatial variation in *KRAS* and *ERBB2* copy number has been observed in a HGSOC resection specimen [19], highlighting a potential limitation to targeting amplified driver genes. However, trunk driver amplifications may be more pervasive and sustained through genomic evolution of HGSOC. Several chromosomal loci are now known to harbour driver genes [2, 15]. 19q12 is one of the best studied loci and contains *CCNE1*, one of the most prevalent and potentially actionable amplified targets in HGSOC [20]. While no agents targeting amplified genes are currently licensed for HGSOC, the success of HER2 targeting agents in treating *ERBB2*-amplified breast and gastric cancers demonstrates the scope for translational research [21]. More recently, short hairpin RNA (shRNA) and CRIPSR screening methods have been employed, providing global and systematic approaches to identify amplified therapeutic targets [18].

This review presents a collation of genes established to be amplified therapeutic targets in HGSOC from the literature, with quantitative appraisal of the supporting evidence. Genes will be scored based on relevant evidence with key experimental strengths and flaws highlighted. Knockdown or knockout of a candidate gene in an OC cell line causing phenotypic suppression was near universal in publications and deemed essential for inclusion, as was evidence of overexpression and gene or locus amplification in human tissue. We devised a scoring system for candidate genes based on cell lines utilised, method of knockdown, breadth of phenotypic characterisation, correlation of expression or amplification to knockdown sensitivity (gene addictivity), evidence of reverse phenotype with gene overexpression, correlation of expression or amplification in human tissue to patient survival, use of in vivo models, use of pharmacological agents, mechanistic interrogation, and any other insightful efforts to validate (see Supplementary Table 1 for scoring system).

## Highest scoring amplified therapeutic targets

Overall, 44 genes were deemed to have sufficient evidence as amplified drivers, including three long non-coding RNAs (lncRNA). The three joint highest scoring genes were *CCNE1*, *PAX8* and *URI1* with scores of 16 out of a theoretical maximum 24. Other genes scoring 9 or higher were *PRCKI*, *FAL1* (lncRNA), *MCL1*, *BCL2L1*, *BCL2*, *ERBB3*, *ID4*, *RSF1* and *CDC42BPA* (see Table 1 for the top scoring genes and Supplementary Table 2 for the full list of genes). The top five scoring genes will be discussed in more detail.

**Table 1:** The 12 highest-scoring amplified therapeutic targets in high-grade serous ovarian carcinoma with appraisal of supporting evidence.

Hazard ratios (HR) and p-values quoted for survival correlation where available. Gene addictivity was defined as cell lines with gene amplification or higher expression showing greater phenotypic suppression on knockdown. Full list of scoring genes and score legend in Supplementary Table 2. CN   copy number, dox-shRNA doxycycline-inducible shRNA, EpCAM epithelial cell adhesion molecule, Exp gene expression, GW   genome-wide, HOSE   human ovarian surface epithelium, IHC  immunohistochemistry, IOSE-M  immortalized ovarian surface epithelial cells, KD   knockdown, OE   overexpression, OS  overall survival, PFS  progression-free survival.

### CCNE1

Cyclin E1, encoded by *CCNE1*, is a key regulator of the cell cycle, promoting G1/S-phase transition mainly in conjunction with cyclin-dependent kinase 2 (CDK2) (ref. [22]). Two papers featuring *CCNE1* knockdown in ovarian cancer cell lines were found. Yang et al. used three small interfering RNA (siRNA) constructs against *CCNE1* in eight OC cell lines. Gene amplification was present in the most cell lines overexpressing *CCNE1*. Only one line was deemed high quality, however the group demonstrated addictivity to *CCNE1*: cell lines overexpressing the gene were more sensitive to siRNA knockdown. Similarly, *CCNE1*-overexpressing lines were more susceptible to the CDK2 inhibitor SNS-032. SNS-032 also increased survival of athymic mice injected with intraperitoneal OCC1 cells, a *CCNE1*-overexpressing cell line, exposing potential translational relevance [22]. Etemadmoghadam et al. performed siRNA knockdown of *CCNE1* on five cell lines, including three high-quality lines. Cell lines with 19q12 amplification were more susceptible to knockdown. Interestingly, knockdown attenuated sensitivity to cisplatin, suggesting combination platinum therapy and CDK2-inhibition may be antagonistic [23]. Demonstration of reverse phenotype to knockdown with induced overexpression is an effective way to support a gene as an oncogenic driver. Karst et al. provided such evidence for *CCNE1* by inducing overexpression in dominant negative *TP53* mutant Fallopian tube secretory epithelial cells (FTSEC) using a viral vector. This increased clonogenicity and anchorage-independent growth, elegantly supporting a role for *CCNE1* in HGSOC carcinogenesis. The group also confirmed correlation of *CCNE1*-amplification in patient tumour tissue with poorer OS, using The Cancer Genome Atlas (TCGA) data [24].

CDK2 inhibition may be viewed as the principal targeted strategy for treatment of *CCNE1*-amplified HGSOC, with SNS-032 (ref. [22]) and dinaciclib [25] having been proposed to this end. SNS-032 has been tested in a phase 1 trial of 20 patients with unselected solid tumours; best response was stable disease (SD) observed in 15% of patients [26]. Dinaciclib has been evaluated in a phase 1 study, which included four patients with OC. Best response was SD [27]. Dinaciclib has also been evaluated in randomised phase 2 studies for treatment of non-small cell lung cancer [28] and breast cancer [29], and has completed phase 3 evaluation for treatment of chronic lymphocytic leukaemia [30]. Fadraciclib, a novel CDK2/CDK9 inhibitor, has undergone pre-clinical evaluation [31] and is currently in early phase clinical trials for advanced solid tumours (NCT04983810). BLU-222, a novel orally bioavailable highly-selective CDK2 inhibitor, is currently undergoing pre-clinical evaluation [32] and is being investigated in early phase clinical trials for advanced solid tumours (NCT05252416).

### PAX8

*PAX8* is a paired box family transcription factor involved in Müllerian tract development and expressed in the Fallopian tube epithelium (FTE) [33]. PAX8 may mediate some tumour-promoting effects through FOXM1 (ref. [34]) and tumour invasiveness through upregulation of FGF18 (ref. [35]). *PAX8* is amplified in 16% of HGSOCs [2], and was a joint-top scoring gene with *CCNE1* in our analysis. Its significance as an amplified therapeutic target was identified by Cheung et al. in a genome-wide shRNA screen performed on a large panel of cell lines, including five high-quality HGSOC lines. In this study, the anti-proliferative effect of *PAX8* knockdown correlated with expression level, and the gene emerged as the highest-ranking dependency in OC cell lines [36]. Hardy et al. demonstrated *PAX8*’s role in cellular migration by performing wound closure and Boyden chamber assays with CRISPR-generated PAX8^−^^/−^ OVCAR4 and OVCAR8 cells. Mouse models revealed reduced proliferation rates in vivo and prolonged survival with *PAX8* knockout. The group demonstrated that the thiopeptide antibiotic thiostrepton reduces PAX8 protein levels by an as yet unidentified mechanism and successfully used micellar thiostrepton to improve survival of mice with OVCAR8 xenografts [34]. Collectively, *PAX8* has strong evidence as an amplified therapeutic target.

Despite micellar thiostrepton being used pre-clinically [34], we could not find publications pertaining to use of thiostrepton or other PAX8 inhibitors in trials. Efforts to identify small molecule inhibitors are underway.

### URI1

URI1 is a member of the prefoldin family of molecular chaperones with roles in apoptotic signalling [37]. Davis et al. sought to identify culprit driver genes in OC by performing an siRNA screen of OC amplicons using 18 cell lines. Four constructs were used per gene target, ensuring good coverage of the amplified genome. Although endometrioid carcinoma cell lines were included, a creditable 11 HGSOC lines were used including four high-quality lines. *URI1*, resides in the 19q12 locus adjacent to *CCNE1*, and was one of the top depleted genes in this robust and systematic study. *URI1* copy number and expression in patient tissue were both found to correlate with OS and PFS from TCGA data [38]. Theurillat et al. found that *URI1* overexpressing cell lines were more sensitive to knockdown than cell lines with low expression and demonstrated that knockdown of *URI1* reduced the in vivo tumorigenicity of *URI1*-amplified OVCAR3 cells. Validating evidence for URI1’s mechanism of effect was also revealed; it increases the threshold for apoptotic death under stress conditions such as growth factor depletion or presence of antitumour drugs such as rapamycin and cisplatin. Overexpressed *URI1* constitutively inhibits PP1γ, reducing the negative feedback of S6K1-BAD survival signalling; a finding corroborated in human ovarian cancer tissue [37].

We could not find clinical trials relevant to *URI1*, or publications pertaining to development of inhibitors.

### PRKCI

*PRKCI* encodes protein kinase C Iota (PKCι), an atypical member of the (serine/threonine) protein kinase C (PKC) family [39]. Although a lesser studied isoform of PKC, it has growing evidence for roles in ovarian cancer biology. *PRKCI* induces YAP1-dependent transformation of FTE cells [39, 40] and promotes an immune-suppressive microenvironment in HGSOC [39]. *PRKCI* overexpression [41] but not amplification [42] is associated with poorer survival. Rehmani et al. provided the key evidence for *PRKCI* as an amplified therapeutic target. They performed an siRNA knockdown in 12 OC lines (three high-quality), demonstrating greater growth inhibition in lines with *PRKCI* amplification. The group also validated an EpCAM aptamer-delivered siRNA as a potential therapeutic using cell line-derived mouse xenograft models [42]. Intraperitoneal auranofin has also been found to reduce the size tumours in mice with OVCAR3 xenografts [40].

The gold compound aurothiomalate is known to inhibit PKCι, and has been evaluated in a phase 1 study, which included four patients with OC. Best response was SD in 13.3% of patients [43]. The compound does not appear to have been evaluated in later-stage clinical trials for cancer. Enzastaurin is a PKC inhibitor which has been evaluated in phase 2 trials for treatment of OC [44]. Enzastaurin in combination with carboplatin and paclitaxel followed by maintenance enzastaurin showed a non-significant trend to improved PFS over carboplatin and paclitaxel alone for treatment of advanced OC in a randomised phase 2 study [45]. We could not find information regarding the potency of enzastaurin towards the PKCι isoform specifically.

### FAL1

Protein-coding sequences occupy less than 2% of the genome [46] and many CNAs in cancer occur in regions devoid of protein-coding potential [5]. Hu et al. aimed to identify oncogenic amplified lncRNAs in ovarian cancer. They validated *FAL1* from a panel of 37 lncRNA genes which were commonly amplified in at least one tumour tissue, commonly overexpressed in cell lines, and located in a focal amplicon. Amplification and overexpression of *FAL1* in OC were associated with poorer OS. siRNA knockdown in a panel of OC cell lines reduced growth and anchorage-independent growth. Ectopic overexpression of the lncRNA induced colony formation in human ovarian surface epithelium (OSE) cells, supporting an oncogenic role for *FAL1*. Intraperitoneal injection of siRNA targeting *FAL1* reduced growth of A2780 xenografts [47]. This supports a potential clinical application to downregulating *FAL1*, however A2780 cells are considered a poor model cell line for HGSOC. *FAL1* was shown to associate with and stabilise BMI1, a known oncoprotein implicated in EOC and key component of the chromatin remodelling polycomb group complex 1 (PRC1) (ref. [48]). *FAL1* regulates transcription of a large number of genes, in part by regulating the interaction of BMI1 with target gene promotor regions [47].

We could not find clinical trials relevant to *FAL1*.

## Methodological approaches and variations

Two papers identifying amplified therapeutic targets employed genome-wide CRISPR screens; both were performed to identify genetic mediators of drug resistance rather than proliferation. Stover et al. performed a CRISPR screen to identify genes mediating cisplatin and paclitaxel resistance on two high-quality cell lines, Kuramochi and OVSAHO, with four BCL2 family genes (*BCL2L1*, *MCL1*, *BCL2* and *BCL2L2*) extensively validated, including with use of BH3-mimetic drugs, such as navitoclax. These genes are amplified in HGSOC, albeit relatively infrequently (4–12%) [49]. While this study may have invaluable clinical potential, no drug-independent phenotypic effects of these genes were identified; these targets may therefore not be as intrinsically relevant to HGSOC biology. *C12orf5* (now referred to as *TIGAR*) was identified from a second genome-wide CRISPR screen, which investigated genes causing olaparib resistance. This was however only performed in A2780 cells [50], limiting the relevance of this study to HGSOC behaviour in vivo. Recently, CRISPR-interference and CRISPR-deletion screens have been employed with RNA sequencing to identify the enriched oncogenic targets of the transcription factor *BRD4* (ref. [51]). Efforts like these can provide highly meaningful insight into the mechanisms by which amplified oncogenic transcription factors mediate their effects and potentially identify downstream targets which may be more amenable to inhibitors.

Several lower ranked genes commonly derived evidence from older studies: use of CRISPR, screening methods and deeper knowledge of cell line quality were not established a decade ago. Some wider studied and acknowledged oncogenes, such as *MYC* and *PIK3CA*, scored perhaps surprisingly low - 6 and 4 respectively. Coming from older bodies of work, the knockdown experiments supporting these genes as amplified therapeutic targets were less robustly performed, e.g., with only a single siRNA construct in both cases [52, 53]. Repeating these studies with multiple shRNAs or generation of CRISPR knockouts in quality cell lines would give a more valid appraisal of these amplified targets.

## Conclusions

In this review, we have quantitatively appraised the evidence supporting genes as amplified therapeutic targets in HGSOC. As clinical investigation of the disease becomes more extensive, copy number analysis of validated oncogenes as standard of care could lead to improved prognostication and tailored treatments. The literature suggests several promising targets. To date however, trials of drugs targeting amplified genes in HGSOC have been disappointing. Interrogation of *ERBB2* amplification in breast cancer has led to therapeutic success, but trials of drugs targeting HER2, including trastuzumab, pertuzumab and lapatinib, have yielded poor results in ovarian cancer [54]. This perhaps reflects the relative paucity of quality pre-clinical evidence supporting *ERBB2* as an amplified therapeutic target in HGSOC (a modest score of 6 in this review). *ERBB2* may have a more limited role in HGSOC carcinogenesis, or may be only a bystander amplification in such cases. The phosphoinositide 3-kinase (PI3K) pathway is one of the most commonly deregulated in OC [2] and *PIK3CA* (encoding the catalytic subunit of PI3K) is amplified in 18% of HGSOCs [25]. Several trials of agents inhibiting nodes of this pathway, including PI3K, have proven disappointing [55, 56]. However, a phase 1 trial of the pan-PI3K inhibitor BKM120 (buparlisib) in combination with olaparib showed some promising responses in unselected OC patients. Interestingly, *PIK3CA* amplifications were not detected in any of the evaluable patients [56]. PI3K pathway inhibition may have a unique niche in sensitising OC to PARP inhibitors, however the impact of relevant gene amplifications to this phenomenon is currently unclear. Poorer responses to single agents targeting this pathway could reflect the lack of quality preclinical evidence supporting *PIK3CA* as an amplified therapeutic target (score 4), the low prevalence of *AKT1*, *AKT2* and *AKT3* amplifications (as downstream nodes of the pathway) in HGSOC [2, 25], or the lack of patient selection by genetic alterations in clinical trials [55]. Further efforts to validate and stratify amplified therapeutic targets will require in silico and in vitro work. More translationally, several papers evaluated in this review suggest that drugs targeting high scoring genes warrant further investigation and consideration of clinical trials.

In our effort to score and rank individual genes as HGSOC amplified targets, limitations to our approach should be acknowledged. Firstly, we could not easily lend weight to prevalence of amplifications due to non-standardised definitions of ‘amplified’. Secondly, in pooled studies, candidate genes may have been quantitatively compared with each other for weight of effect, however these comparisons are difficult to merit with differential scores due to breadth of work and experimental techniques. Lastly, standard in vitro knockdown experiments are unable to reflect the influence of amplified driver gene signalling on the tumour microenvironment (TME) [57], which may limit the direct clinical relevance of these studies.

## Perspectives and future research

### In silico

Conventional methods, including siRNA screens and shRNA/siRNA targeting single genes to investigate functions of amplified genes are limited by relative low-throughput and off-target effects. Recent advances in whole genome functional genomics including CRISPR/Cas9 and pooled shRNA screens have produced robust and high-throughput data. Project Achilles is an ongoing shRNA and CRISPR/Cas9 screening effort, now incorporating over 700 cancer cell lines, integrated with CNA and other genomic data [58]. In parallel, Project Score has performed CRISPR/Cas9 screens for over 900 cancer cell lines with genomic and drug screen data [59]. These efforts provide unique opportunities to systematically analyse the functional impact of amplified genes in CNDCs. In addition, existing publicly available data from TCGA and International Cancer Genome Consortium (ICGC) studies provide a fast and robust means to study prevalence and clinical impact of amplified genes [60]. Gene amplifications not known to correlate with OS outcomes can be assessed for their impact in an unbiased fashion using such repositories, as well as PFS or disease-free survival (DFS), the latter two of which were not as frequently observed in this literature search. Future works to combine these efforts will potentially identify a more comprehensive and clinically relevant list of amplified therapeutic targets.

### In vitro

Several genes scored relatively low in our analysis due to experimental use of poor-quality cell lines. Knockdown studies for *PIK3CA*, *PI3*, *ERBB3*, *ERBB2*, *FOXM1* and *SKIL* in a large panel of high-quality cell lines will give a better representation of their oncogenic roles. Several prominent oncogenes such as *KRAS*, *TERT*, *AKT1* and *AKT3*, were not found to have sufficient evidence as amplified therapeutic targets from our secondary search. A systemic investigation of such known oncogenes from other cancer types may be required for HGSOC specifically.

HGSOC is characterised by an immunosuppressive TME, attributed to low tumour mutational burden with consequent low neo-antigen expression, epigenetic silencing of Th1-cytokines, and tumour endothelial Fas ligand and endothelin B receptor expression [61]. While *PRKCI* is implicated in HGSOC immune suppression [39], the significance of gene amplifications in mediating tumour immune escape is largely elusive. Knockdown studies performed on co-cultures of HGSOC cells with immune cells (cytotoxic T cells, myeloid-derived suppressor cells, macrophages, NK cells or neutrophils) could help to identify drivers that function more in the context of TME contribution. The contribution of amplified therapeutic targets to oncogenic signalling with stromal cells, e.g., cancer-associated fibroblasts, mesenchymal stem cells and endothelial cells, also warrants investigation given their emerging role in the disease [62, 63].

Intratumoral heterogeneity is likely to represent a major challenge in the development of HGSOC therapeutics. Clonal evolution yielding variations in chromosome and gene copy number occur through disease progression and following treatment with chemotherapy [19, 64]. However, understanding of spatial and temporal changes in CNAs of given driver genes is limited, and may be important in selecting the most clinically promising targets. Single cell DNA sequencing has been employed to interrogate overall copy number heterogeneity within primary and metastatic tumour deposits; interestingly in two analysed patients with HGSOC, less heterogeneity was observed in metastatic deposits than the paired primary tumours [65]. Studies of copy number heterogeneity for given amplified therapeutic targets should be considered, which can be performed in parallel with transcriptomic studies to appraise the influence of the TME on their expression. Challenges to research in this area include the difficulty and risk of obtaining tumour samples for multiple metastases, and the need for repeat biopsies to study temporal evolution. Circulating tumour cells may offer a simplified means to study temporal, but not spatial clonal evolution of amplified therapeutic targets [66].

### Drug development and clinical trials

Evidence is mounting that several established drugs may be of benefit in HGSOCs with the relevant gene amplification. SNS-032 and dinaciclib have undergone clinical evaluation, however specific activity in *CCNE1*-amplified or overexpressing HGSOC does not appear to have been investigated and could be considered in phase 2/3 trials. Fadraciclib could prove a more efficacious agent given its greater CDK2 selectivity [31]. BLU-222 in particular, as a highly-selective orally bioavailable CDK2 inhibitor, could ultimately demonstrate even greater potential in this setting following confirmation of safety in early phase trials [32]. *CCNE1*-amplification may also yield other therapeutic vulnerabilities. Wee1, a serine-threonine kinase, inhibits CDK1 and CDK2 in response to DNA damage, thereby halting G2 and G1/S phase cell cycle progression respectively. Cancers often depend on the G2-M checkpoint to prevent mitotic catastrophe, hence Wee1 inhibitors can induce cancer cell apoptosis. Wee1 inhibition also enhances detection of DNA damage at G2/M phase [67]. Adavosertib, a selective Wee1 inhibitor has shown promise in an early-phase trial in solid tumours; one patient with *CCNE1-*amplification and one with overexpression responded to therapy, while *CCNE1* overexpression was not seen in non-responders [67]. Pre-clinical models of triple-negative breast cancer also suggest *CCNE1* overexpression predicts sensitivity to adavosertib [68]. The relevance of *CCNE1* amplifications to Wee1 inhibition should be further explored with both pre-clinical and clinical studies. Given their somewhat opposing effects on the cell cycle, CDK2 and Wee1 inhibition may not be synergistic, however this could be first explored with in vitro cytotoxicity studies. *CDK2* is amplified in 6.4% of ovarian tumours; [69] although not validated as an amplified therapeutic target from our literature search, this may present a similar therapeutic vulnerability. Also within the top five scoring amplified therapeutic targets, *PRKCI* may be vulnerable to existing compounds. Selective small molecule PKCι inhibitors such as ICA-1 have been investigated in prostate cancer cell lines [70]. Following evaluation on relevant HGSOC cell lines, such compounds should be considered for pharmacokinetic and early human studies of *PRKCI*-amplified HGSOC.

Other high-scoring amplified therapeutic targets may be amenable to existing agents which can be explored with well-designed clinical trials. Seribantumab, a HER3-targeting monoclonal antibody, was evaluated in combination with paclitaxel in a phase 2 trial for platinum resistant or refractory OC, although did not improve the endpoint of PFS [71]. This agent could be further developed with selection by biomarkers (e.g., *ERBB3* amplification) in phase 2 studies. Development of antibody-drug conjugates and small molecule inhibitors targeting HER3 can also be considered. Several BCL2 family genes scored highly, and BH3-mimetics have therefore been proposed as treatments, particularly in the context of synergising with chemotherapy or re-sensitising resistant cancers to chemotherapy: [49] in vivo combination cytotoxicity studies may help to refine clinical trials for relapsed disease. *CDC42BPA*, encoding MRCKA, scored 9 in this study. MRCKA was successfully targeted in vitro with the small molecule inhibitor BDP9066 (ref. [72]). This agent could be developed further towards clinical trials. *PRLR*, encoding the prolactin receptor, scored 8; agents inhibiting secretion of prolactin and receptor antagonists could be considered for clinical development in HGSOC. *AKT2* scored 8 in this review. AKT inhibitors are under development for treatment of various cancers and other nodes of the PI3K-AKT pathway are also clinically targeted. Development of selective inhibitors for *AKT2*-amplified HGSOC may enhance clinical utility by reducing off-target effects.

Given the number of high-scoring targets which are deemed either ‘undruggable’ or difficult to drug, the development of RNAi or even CRISPR/Cas9 based therapeutics against such oncogenes should be explored. This is an appealing direction given recent successes in the clinic for RNA therapies, including covid-19 vaccines and patisiran, an siRNA therapeutic for hereditary transthyretin-mediated amyloidosis, both utilising lipid nanoparticle vectors [73]. Liposomal vectors for cancer RNA therapies are also showing promising preliminary clinical data [74]. *PAX8*, *URI1* and *FAL1* may be the most lucrative targets to explore with such approaches given their strong supportive evidence, however more druggable targets can also be considered.

A final clinical consideration, beyond validation of single-drug approaches to individual amplified therapeutic targets, is combining such agents with existing therapies, as well as eventually other prospective agents in a strategic manner. Pre-clinical studies are an important first step to this end since inhibitors of amplified therapeutic targets may be cytostatic and therefore potentially antagonistic with conventional cytotoxic drugs such as paclitaxel [75]. This is however likely to be highly dependent on the individual amplified gene’s function, and the opportunity for therapeutic synergy ultimately needs to be explored. Given their recent clinical successes in HGSOC, PARP inhibitors may be a lucrative class of drugs to test in combination with agents targeting amplified driver oncogenes. Indeed, pre-clinical evidence suggests cell cycle blockade and PARP inhibition may be synergistic [31, 76]. Finally, inhibition of certain amplified therapeutic targets may also render the cancer more vulnerable to immunotherapy approaches. CDK2 inhibition in mouse models of triple-negative breast cancer has been shown to increase susceptibility to PD-L1 blockade [77]. Inhibition of other amplified targets may also increase immunogenicity, for instance via epigenetic mechanisms.

With sluggish progress in drug approvals and many patients still fated to poor prognoses, HGSOC is a disease in urgent need of superior therapeutics. Given the heterogeneity of the disease, successes are likely to depend on tailoring treatments to the individual; identifying amplified therapeutic targets represents a logical step forward in this respect. In this literature review we have collated and appraised the genes which drive HGSOC carcinogenesis through amplification to give insight into the genomic disorder underpinning the disease and potential avenues for drug development.

## Methods

We initially conducted a PubMed search of the Medline database using the following search terms: (ovarian cancer) AND ((gene) OR (target)) AND ((driver) OR (addicted) OR (amplified)) AND ((rna interference) OR (shrna) OR (sirna) OR (crispr)). This yielded 93 results (as of 17th April 2022). Essential criteria for inclusion were:

1. Research interrogates OC non-selectively or HGSOC (other subtypes excluded)
2. Knockdown experiments are performed on at least one human OC cell line
3. Gene of interest is either known or demonstrated to be overexpressed in human OC tissue
4. Gene or gene locus is either known or demonstrated to be amplified in human OC tissue

Where a criterion was not evidenced in a paper, a basic PubMed search of the Medline database was conducted to identify if this had been established elsewhere. Thirty papers studying 36 genes met these criteria; 63 papers were excluded. A further search was conducted for each gene of interest to collate additional evidence where this was lacking from the hits of the primary search. An additional search was conducted for some well-established oncogenes which did not appear in papers from the primary search, which added a further 8 genes. These were likely missed from the primary search because more historical work in this field was less integrated and, where these genes have been identified from screening techniques, researchers may favour novel genes for validation. This supplementary search is noted as a potential source of bias in this review but needed to avoid omission of key genes.

### Scoring criteria

See Supplementary Table 1 for the full gene scoring criteria. We lent a large score weight to the choice of cell lines used for knockdown studies. EOC encompasses distinct histological subtypes with different mutational and copy number landscapes, leading to different vulnerabilities. Mounting evidence suggests HGSOC arises from the FTE rather than the OSE, which may be distinct from other OC subtypes [20]. The more representative a cell line is of HGSOC, the more clinically relevant the work becomes. Additionally, use of larger panels of cell lines increases experimental validity [38].

The availability of public datasets has permitted interrogation of cell line quality: in 2013, Domcke et al. performed a genomic comparison of patient specimens in TCGA repository and cell lines from the Cancer Cell Line Encyclopedia (CCLE). As well as identifying several high-quality HGSOC model cell lines, they revealed that some commonly used cell lines are poorly representative. SKOV3 and A2780 were then the two most frequently used cell lines for OC research: both are *TP53* wildtype, both have *ARID1A* and *PIK3CA* mutations and both have poor CNA correlation with HGSOC, implying they were more likely derived from clear cell or endometrioid cancers and diminishing their validity as HGSOC models. As a further example, IGROV1 is hypermutated compared to patient HGSOC tissue [78]. We initially stratified cell lines into high, intermediate and low quality based primarily on this paper. Where cell lines which were not reported in this paper were used, they were classed as intermediate by default, and demoted to low quality if they were *TP53* wildtype, derived from a non-HGSOC subtype, wrong tissue of origin, or non-human.

The next experimental factor in knockdown studies is method of knockdown. RNA interference (RNAi) involves neutralisation of specific messenger RNA (mRNA) leading to reduced translation. This can be performed experimentally with siRNA and shRNA, both of which offer greater activity than antisense oligonucleotides (ASO). While siRNA can be synthesised and administered exogenously, shRNA requires nuclear expression through a vector (e.g., lentivirus). Due to continuous expression, shRNA gives a more stable and durable knockdown [79]. Conversely, CRIPSR-Cas9 induces insertion-deletion mutations into the genome at sites corresponding to the guide RNA in the vector construct, abolishing production of active gene transcript. CRISPR is therefore deemed a more robust method of knockdown once on-target effect is confirmed, with fewer off-target effects still [18]. As such, CRISPR screening with pooled or arrayed vectors is a particularly powerful tool in studying cancer drivers. One caveat to CRISPR is that it can generate false positive hits in highly amplified non-driver regions by inducing multiple double stranded breaks, leading to apoptosis. Validation of CRISPR screen results with shRNA knockdown could be viewed as the gold standard of knockdown study [18], and we reflected this with our scoring criteria.

The next consideration in knockdown studies is the phenotype(s) being investigated. The most common in this review was cell growth or apoptosis, measured with various assays. Other cancer phenotypes investigated included migration and invasiveness, metabolism, drug sensitivity and anchorage independence/anoikis escape. Proof of a driver’s role in multiple phenotypes was rewarded an additional score. Demonstrating increased phenotypic suppression with knockdown in amplified or overexpressing cell lines relative to unamplified or lower expressing cell lines adds weight to an amplified oncogene being a driver; it implies dependence on or addiction to that gene [22]. Another key piece of evidence supporting knockdown studies is demonstration of reverse phenotype, i.e., overexpression of the gene of interest generating malignant behaviour. FTE cells or FTSECs are the ideal cells to model reverse phenotype, as they are now accepted as the cell of origin for HGSOC, but OSE cells have also been used [20].

Clinical evidence supporting a gene as a driver is also valuable. If gene amplification is contributing to malignant phenotype, poorer patient survival might be expected when this is present [24, 38]. Additional score weight was added for correlation of driver gene amplification or expression to OS, PFS or DFS in patient datasets. As animal models represent the major bridge between in vitro studies and clinical studies [80], we also gave genes an additional score where knockdown experiments were successfully replicated in animal models. Finally, successful use of pharmaceuticals was given credit as it infers translational potential.

## Supplementary information

Supplementary Table 1

Supplementary Table 2

References for Supplementary Material

## Data Availability

All data generated or analysed during this study are included in this published article [and its supplementary information files].
