# Supplementary material for: Amplified therapeutic targets in high-grade serous ovarian carcinoma – a review of the literature with quantitative appraisal
Source: Cancer Gene Ther. 2023 Feb 20;30(7):955–63. doi: 10.1038/s41417-023-00589-z (PMC9940086; doi:10.1038/s41417-023-00589-z)
Supplement: Supplementary file 1 — Supplementary Table 1 [file 41417_2023_589_MOESM1_ESM.docx]

**Supplementary Table 1.** Scoring criteria and cell lines.


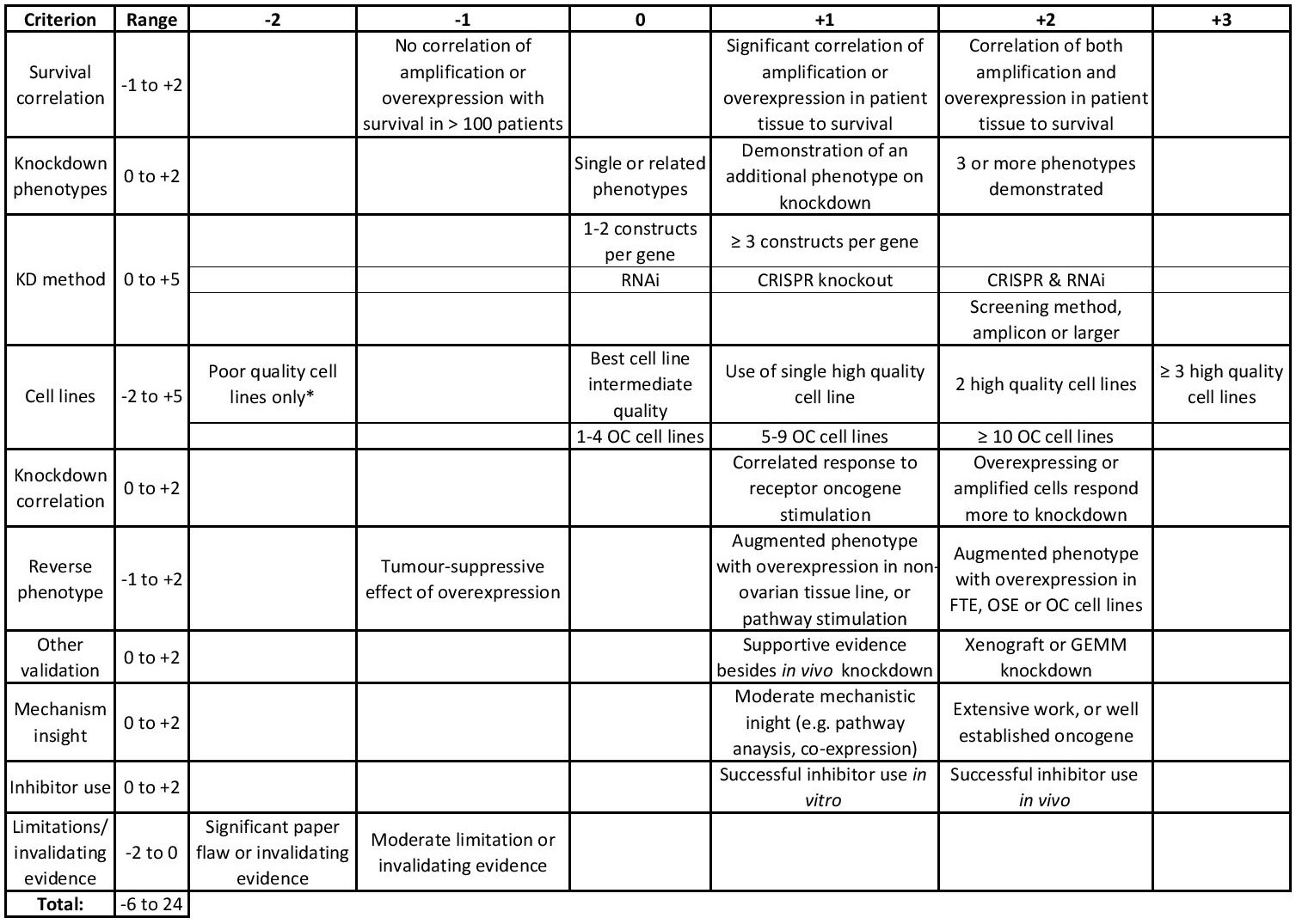


*Negative scores for cell lines only applied if offsetting a score generating experiment from the same paper

GEMM = Genetically engineered mouse model

**High quality cell lines:**

KURAMOCHI, (NIH)OVCAR3, OVCAR4, CaOV3, CaOV4, FUOV1^(63)^

**Intermediate cell lines:**

OVCAR8, OV90, ES-2^(63)^

Cell lines with limited data – OVCA420, OV1847, COV362, OVCAR10

**Poor quality cell lines:**

A2780, SKOV3, HEY-A8, OC316, IGROV1^(63)^

Other *TP53* wildtype lines: HEY, OVCA429^(64)^

HeLa, OVCAR5 (GI origin)^(65)^

OCCC lines (excluding ES-2), low grade serous cell lines and mucinous cell lines (as per ExPaSy Cellosaurus)^(66)^
