# Supplementary material for: Amplified therapeutic targets in high-grade serous ovarian carcinoma – a review of the literature with quantitative appraisal
Source: Cancer Gene Ther. 2023 Feb 20;30(7):955–63. doi: 10.1038/s41417-023-00589-z (PMC9940086; doi:10.1038/s41417-023-00589-z)
Supplement: Supplementary file 3 — References for Supplementary Material [file 41417_2023_589_MOESM3_ESM.docx]

**References for supplementary material**

1. Yang L, Fang D, Chen H, Lu Y, Dong Z, Ding HF, et al. Cyclin-dependent kinase 2 is an ideal target for ovary tumors with elevated cyclin E1 expression. Oncotarget. 2015;6(25):20801-12.

2. Etemadmoghadam D, George J, Cowin PA, Cullinane C, Kansara M, Australian Ovarian Cancer Study G, et al. Amplicon-dependent CCNE1 expression is critical for clonogenic survival after cisplatin treatment and is correlated with 20q11 gain in ovarian cancer. PLoS One. 2010;5(11):e15498.

3. Karst AM, Jones PM, Vena N, Ligon AH, Liu JF, Hirsch MS, et al. Cyclin E1 deregulation occurs early in secretory cell transformation to promote formation of fallopian tube-derived high-grade serous ovarian cancers. Cancer Res. 2014;74(4):1141-52.

4. Mhawech-Fauceglia P, Wang D, Samrao D, Godoy H, Ough F, Liu S, et al. Pair Box 8 (PAX8) protein expression in high grade, late stage (stages III and IV) ovarian serous carcinoma. Gynecol Oncol. 2012;127(1):198-201.

5. Hardy LR, Pergande MR, Esparza K, Heath KN, Onyuksel H, Cologna SM, et al. Proteomic analysis reveals a role for PAX8 in peritoneal colonization of high grade serous ovarian cancer that can be targeted with micelle encapsulated thiostrepton. Oncogene. 2019;38(32):6003-16.

6. Cheung HW, Cowley GS, Weir BA, Boehm JS, Rusin S, Scott JA, et al. Systematic investigation of genetic vulnerabilities across cancer cell lines reveals lineage-specific dependencies in ovarian cancer. Proc Natl Acad Sci U S A. 2011;108(30):12372-7.

7. Theurillat JP, Metzler SC, Henzi N, Djouder N, Helbling M, Zimmermann AK, et al. URI is an oncogene amplified in ovarian cancer cells and is required for their survival. Cancer Cell. 2011;19(3):317-32.

8. Davis SJ, Sheppard KE, Pearson RB, Campbell IG, Gorringe KL, Simpson KJ. Functional analysis of genes in regions commonly amplified in high-grade serous and endometrioid ovarian cancer. Clin Cancer Res. 2013;19(6):1411-21.

9. Sarkar S, Bristow CA, Dey P, Rai K, Perets R, Ramirez-Cardenas A, et al. PRKCI promotes immune suppression in ovarian cancer. Genes Dev. 2017;31(11):1109-21.

10. Wang Y, Justilien V, Brennan KI, Jamieson L, Murray NR, Fields AP. PKCiota regulates nuclear YAP1 localization and ovarian cancer tumorigenesis. Oncogene. 2017;36(4):534-45.

11. Weichert W, Gekeler V, Denkert C, Dietel M, Hauptmann S. Protein kinase C isoform expression in ovarian carcinoma correlates with indicators of poor prognosis. Int J Oncol. 2003;23(3):633-9.

12. Rehmani H, Li Y, Li T, Padia R, Calbay O, Jin L, et al. Addiction to protein kinase Ci due to PRKCI gene amplification can be exploited for an aptamer-based targeted therapy in ovarian cancer. Signal Transduct Target Ther. 2020;5(1):140.

13. Hu X, Feng Y, Zhang D, Zhao SD, Hu Z, Greshock J, et al. A functional genomic approach identifies FAL1 as an oncogenic long noncoding RNA that associates with BMI1 and represses p21 expression in cancer. Cancer Cell. 2014;26(3):344-57.

14. Cancer Genome Atlas Research N. Integrated genomic analyses of ovarian carcinoma. Nature. 2011;474(7353):609-15.

15. Sheng Q, Liu X, Fleming E, Yuan K, Piao H, Chen J, et al. An activated ErbB3/NRG1 autocrine loop supports in vivo proliferation in ovarian cancer cells. Cancer Cell. 2010;17(3):298-310.

16. Tanner B, Hasenclever D, Stern K, Schormann W, Bezler M, Hermes M, et al. ErbB-3 predicts survival in ovarian cancer. J Clin Oncol. 2006;24(26):4317-23.

17. Stover EH, Baco MB, Cohen O, Li YY, Christie EL, Bagul M, et al. Pooled Genomic Screens Identify Anti-apoptotic Genes as Targetable Mediators of Chemotherapy Resistance in Ovarian Cancer. Mol Cancer Res. 2019;17(11):2281-93.

18. Williams J, Lucas PC, Griffith KA, Choi M, Fogoros S, Hu YY, et al. Expression of Bcl-xL in ovarian carcinoma is associated with chemoresistance and recurrent disease. Gynecol Oncol. 2005;96(2):287-95.

19. Shigemasa K, Katoh O, Shiroyama Y, Mihara S, Mukai K, Nagai N, et al. Increased MCL-1 expression is associated with poor prognosis in ovarian carcinomas. Jpn J Cancer Res. 2002;93(5):542-50.

20. Mano Y, Kikuchi Y, Yamamoto K, Kita T, Hirata J, Tode T, et al. Bcl-2 as a predictor of chemosensitivity and prognosis in primary epithelial ovarian cancer. Eur J Cancer. 1999;35(8):1214-9.

21. Ren Y, Cheung HW, von Maltzhan G, Agrawal A, Cowley GS, Weir BA, et al. Targeted tumor-penetrating siRNA nanocomplexes for credentialing the ovarian cancer oncogene ID4. Sci Transl Med. 2012;4(147):147ra12.

22. Yang YI, Ahn JH, Lee KT, Shih Ie M, Choi JH. RSF1 is a positive regulator of NF-kappaB-induced gene expression required for ovarian cancer chemoresistance. Cancer Res. 2014;74(8):2258-69.

23. Shih Ie M, Sheu JJ, Santillan A, Nakayama K, Yen MJ, Bristow RE, et al. Amplification of a chromatin remodeling gene, Rsf-1/HBXAP, in ovarian carcinoma. Proc Natl Acad Sci U S A. 2005;102(39):14004-9.

24. Choi JH, Sheu JJ, Guan B, Jinawath N, Markowski P, Wang TL, et al. Functional analysis of 11q13.5 amplicon identifies Rsf-1 (HBXAP) as a gene involved in paclitaxel resistance in ovarian cancer. Cancer Res. 2009;69(4):1407-15.

25. Kurimchak AM, Herrera-Montavez C, Brown J, Johnson KJ, Sodi V, Srivastava N, et al. Functional proteomics interrogation of the kinome identifies MRCKA as a therapeutic target in high-grade serous ovarian carcinoma. Sci Signal. 2020;13(619).

26. Karthikeyan S, Russo A, Dean M, Lantvit DD, Endsley M, Burdette JE. Prolactin signaling drives tumorigenesis in human high grade serous ovarian cancer cells and in a spontaneous fallopian tube derived model. Cancer Lett. 2018;433:221-31.

27. Au-Yeung G, Lang F, Azar WJ, Mitchell C, Jarman KE, Lackovic K, et al. Selective Targeting of Cyclin E1-Amplified High-Grade Serous Ovarian Cancer by Cyclin-Dependent Kinase 2 and AKT Inhibition. Clin Cancer Res. 2017;23(7):1862-74.

28. Linnerth-Petrik NM, Santry LA, Moorehead R, Jucker M, Wootton SK, Petrik J. Akt isoform specific effects in ovarian cancer progression. Oncotarget. 2016;7(46):74820-33.

29. Zheng B, Geng L, Zeng L, Liu F, Huang Q. AKT2 contributes to increase ovarian cancer cell migration and invasion through the AKT2-PKM2-STAT3/NF-kappaB axis. Cell Signal. 2018;45:122-31.

30. Fang P, De Souza C, Minn K, Chien J. Genome-scale CRISPR knockout screen identifies TIGAR as a modifier of PARP inhibitor sensitivity. Commun Biol. 2019;2:335.

31. Simpkins FA, Devoogdt NM, Rasool N, Tchabo NE, Alejandro EU, Kamrava MM, et al. The alarm anti-protease, secretory leukocyte protease inhibitor, is a proliferation and survival factor for ovarian cancer cells. Carcinogenesis. 2008;29(3):466-72.

32. Park JT, Li M, Nakayama K, Mao TL, Davidson B, Zhang Z, et al. Notch3 gene amplification in ovarian cancer. Cancer Res. 2006;66(12):6312-8.

33. Kang H, Jeong JY, Song JY, Kim TH, Kim G, Huh JH, et al. Notch3-specific inhibition using siRNA knockdown or GSI sensitizes paclitaxel-resistant ovarian cancer cells. Mol Carcinog. 2016;55(7):1196-209.

34. Liu Z, Yun R, Yu X, Hu H, Huang G, Tan B, et al. Overexpression of Notch3 and pS6 Is Associated with Poor Prognosis in Human Ovarian Epithelial Cancer. Mediators Inflamm. 2016;2016:5953498.

35. Park JT, Chen X, Trope CG, Davidson B, Shih Ie M, Wang TL. Notch3 overexpression is related to the recurrence of ovarian cancer and confers resistance to carboplatin. Am J Pathol. 2010;177(3):1087-94.

36. Choudhury A, Charo J, Parapuram SK, Hunt RC, Hunt DM, Seliger B, et al. Small interfering RNA (siRNA) inhibits the expression of the Her2/neu gene, upregulates HLA class I and induces apoptosis of Her2/neu positive tumor cell lines. Int J Cancer. 2004;108(1):71-7.

37. Chung YW, Kim S, Hong JH, Lee JK, Lee NW, Lee YS, et al. Overexpression of HER2/HER3 and clinical feature of ovarian cancer. J Gynecol Oncol. 2019;30(5):e75.

38. Wang W, Gao Y, Hai J, Yang J, Duan S. HER2 decreases drug sensitivity of ovarian cancer cells via inducing stem cell-like property in an NFkappaB-dependent way. Biosci Rep. 2019;39(3).

39. Zhao F, Siu MK, Jiang L, Tam KF, Ngan HY, Le XF, et al. Overexpression of forkhead box protein M1 (FOXM1) in ovarian cancer correlates with poor patient survival and contributes to paclitaxel resistance. PLoS One. 2014;9(11):e113478.

40. Barger CJ, Zhang W, Hillman J, Stablewski AB, Higgins MJ, Vanderhyden BC, et al. Genetic determinants of FOXM1 overexpression in epithelial ovarian cancer and functional contribution to cell cycle progression. Oncotarget. 2015;6(29):27613-27.

41. Westhoff GL, Chen Y, Teng NNH. Targeting Foxm1 Improves Cytotoxicity of Paclitaxel and Cisplatinum in Platinum-Resistant Ovarian Cancer. Int J Gynecol Cancer. 2017;27(5):887-94.

42. Prathapam T, Aleshin A, Guan Y, Gray JW, Martin GS. p27Kip1 mediates addiction of ovarian cancer cells to MYCC (c-MYC) and their dependence on MYC paralogs. J Biol Chem. 2010;285(42):32529-38.

43. Jung M, Russell AJ, Kennedy C, Gifford AJ, Australian Ovarian Cancer Study G, Mallitt KA, et al. Clinical Importance of Myc Family Oncogene Aberrations in Epithelial Ovarian Cancer. JNCI Cancer Spectr. 2018;2(3):pky047.

44. Karst AM, Levanon K, Drapkin R. Modeling high-grade serous ovarian carcinogenesis from the fallopian tube. Proc Natl Acad Sci U S A. 2011;108(18):7547-52.

45. Clauss A, Ng V, Liu J, Piao H, Russo M, Vena N, et al. Overexpression of elafin in ovarian carcinoma is driven by genomic gains and activation of the nuclear factor kappaB pathway and is associated with poor overall survival. Neoplasia. 2010;12(2):161-72.

46. Labidi-Galy SI, Clauss A, Ng V, Duraisamy S, Elias KM, Piao HY, et al. Elafin drives poor outcome in high-grade serous ovarian cancers and basal-like breast tumors. Oncogene. 2015;34(3):373-83.

47. Ding Y, Fang Q, Li Y, Wang Y. Amplification of lncRNA PVT1 promotes ovarian cancer proliferation by binding to miR-140. Mamm Genome. 2019;30(7-8):217-25.

48. Guan Y, Kuo WL, Stilwell JL, Takano H, Lapuk AV, Fridlyand J, et al. Amplification of PVT1 contributes to the pathophysiology of ovarian and breast cancer. Clin Cancer Res. 2007;13(19):5745-55.

49. Hagerstrand D, Tong A, Schumacher SE, Ilic N, Shen RR, Cheung HW, et al. Systematic interrogation of 3q26 identifies TLOC1 and SKIL as cancer drivers. Cancer Discov. 2013;3(9):1044-57.

50. George J, Li Y, Kadamberi IP, Parashar D, Tsaih SW, Gupta P, et al. RNA-binding protein FXR1 drives cMYC translation by recruiting eIF4F complex to the translation start site. Cell Rep. 2021;37(5):109934.

51. Mazzoldi EL, Pasto A, Ceppelli E, Pilotto G, Barbieri V, Amadori A, et al. Casein Kinase 1 Delta Regulates Cell Proliferation, Response to Chemotherapy and Migration in Human Ovarian Cancer Cells. Front Oncol. 2019;9:1211.

52. Pongas G, Kim MK, Min DJ, House CD, Jordan E, Caplen N, et al. BRD4 facilitates DNA damage response and represses CBX5/Heterochromatin protein 1 (HP1). Oncotarget. 2017;8(31):51402-15.

53. Karmakar S, Seshacharyulu P, Lakshmanan I, Vaz AP, Chugh S, Sheinin YM, et al. hPaf1/PD2 interacts with OCT3/4 to promote self-renewal of ovarian cancer stem cells. Oncotarget. 2017;8(9):14806-20.

54. Nanjundan M, Cheng KW, Zhang F, Lahad J, Kuo WL, Schmandt R, et al. Overexpression of SnoN/SkiL, amplified at the 3q26.2 locus, in ovarian cancers: a role in ovarian pathogenesis. Mol Oncol. 2008;2(2):164-81.

55. Cheng KW, Lahad JP, Kuo WL, Lapuk A, Yamada K, Auersperg N, et al. The RAB25 small GTPase determines aggressiveness of ovarian and breast cancers. Nat Med. 2004;10(11):1251-6.

56. Zhang L, Yang N, Liang S, Barchetti A, Vezzani C, Huang J, et al. RNA interference: a potential strategy for isoform-specific phosphatidylinositol 3-kinase targeted therapy in ovarian cancer. Cancer Biol Ther. 2004;3(12):1283-9.

57. Kolasa IK, Rembiszewska A, Felisiak A, Ziolkowska-Seta I, Murawska M, Moes J, et al. PIK3CA amplification associates with resistance to chemotherapy in ovarian cancer patients. Cancer Biol Ther. 2009;8(1):21-6.

58. Ediriweera MK, Tennekoon KH, Samarakoon SR. Role of the PI3K/AKT/mTOR signaling pathway in ovarian cancer: Biological and therapeutic significance. Semin Cancer Biol. 2019;59:147-60.

59. Sethi G, Pathak HB, Zhang H, Zhou Y, Einarson MB, Vathipadiekal V, et al. An RNA interference lethality screen of the human druggable genome to identify molecular vulnerabilities in epithelial ovarian cancer. PLoS One. 2012;7(10):e47086.

60. Liu Q, Liu H, Li L, Dong X, Ru X, Fan X, et al. ATAD2 predicts poor outcomes in patients with ovarian cancer and is a marker of proliferation. Int J Oncol. 2020;56(1):219-31.

61. Gordon MA, Babbs B, Cochrane DR, Bitler BG, Richer JK. The long non-coding RNA MALAT1 promotes ovarian cancer progression by regulating RBFOX2-mediated alternative splicing. Mol Carcinog. 2019;58(2):196-205.

62. O'Brien PM, Davies MJ, Scurry JP, Smith AN, Barton CA, Henderson MJ, et al. The E3 ubiquitin ligase EDD is an adverse prognostic factor for serous epithelial ovarian cancer and modulates cisplatin resistance in vitro. Br J Cancer. 2008;98(6):1085-93.

63. Domcke S, Sinha R, Levine DA, Sander C, Schultz N. Evaluating cell lines as tumour models by comparison of genomic profiles. Nat Commun. 2013;4:2126.

64. Watson JL, Greenshields A, Hill R, Hilchie A, Lee PW, Giacomantonio CA, et al. Curcumin-induced apoptosis in ovarian carcinoma cells is p53-independent and involves p38 mitogen-activated protein kinase activation and downregulation of Bcl-2 and survivin expression and Akt signaling. Mol Carcinog. 2010;49(1):13-24.

65. Blayney JK, Davison T, McCabe N, Walker S, Keating K, Delaney T, et al. Prior knowledge transfer across transcriptional data sets and technologies using compositional statistics yields new mislabelled ovarian cell line. Nucleic Acids Res. 2016;44(17):e137.

66. Duvaud S, Gabella C, Lisacek F, Stockinger H, Ioannidis V, Durinx C. Expasy, the Swiss Bioinformatics Resource Portal, as designed by its users. Nucleic Acids Res. 2021.
